# Department of Practice of Medicine

**Published:** 1894-02

**Authors:** 

**Affiliations:** Galveston, Professor of Pathology and Lecturer on Mental and Nervous Diseases in the Medical Department of the University of Texas, Galveston


### Current Medical Literature.

### DEPARTMENT OF PRACTICE OF MEDICINE.

EDITED BY PROF. ALLEN J. SMITH, M. D., GALVESTON,
Professor of Pathology and Lecturer on Mental and Nervous Diseases in the Medical Department of the University of Texas, Galveston.

PREVENTIVE INOCULATIONS AGAINST HYDROPHOBIA AT THE CHICAGO PASTEUR INSTITUTE.—Dr. A. Lagorio, Director of the Chicago Pasteur Institute (*St. Louis Clinique*, Dec., 1893), gives the following as the results attained at the Institute since its inauguration on the second of July, 1890: Three hundred and two cases have been treated to date. Of these one hundred and four were individuals bitten by animals fully ascertained to have been rabid by laboratory experiments, or by the death of other persons or animals bitten; one hundred and twenty-six were bitten by animals recognized as mad by the symptoms shown during life; seventy-two were bitten by animals strongly suspected as being rabid. Two hundred and eighty-two were bitten by dogs; seven by horses; seven by cats; three by skunks; two by wolves; one by a mule. The patients were distributed as follows among the different States: One hundred and eighty-five from Illinois; thirty-two from Iowa; twenty-three from Indiana; twenty-one from Kansas; nine from Ohio; five from Missouri; five from Arizona; four from Minnesota; four from Michigan; four from Louisiana; two from Texas; one each from Wisconsin and South Dakota.

Only one death (0.33 per cent) is known to have occurred among these patients.

---

THE DIAZO-REACTION OF EHRLICH.—(*N. Y. Med. Jour.*, Dec. 23, 1893.) Dr. Julius Friedenwald, of Baltimore, has recently published the records of a large number of examinations of the urine in cases of typhoid fever for the diazo-reaction of Ehrlich. Ehrlich, in announcing this test, claimed for it marked diagnostic value in case of typhoid fever; that it is most commonly obtained from the middle of the first week of illness; that when it is missing, the diagnosis of typhoid fever is doubtful; that where it is slightly shown the cases are usually very mild; that when the reaction is met in cases of pulmonary phthisis it is of very grave prognostic significance; that it is sometimes, but rarely, met in cases of measles, miliary tuberculosis, piæmia, scarlet fever, and erysipelas; that in all non-febrile diseases as chlorosis, hydræmia, diabetes, brain-, spinal-, liver- or kidney-disease, the reaction is never obtained. Friedenwald holds that where the conclusions of Ehrlich have been contradicted by authors who claim the failure of value in the reaction because of its occurrence in a variety of febrile and non-febrile diseases, that it has not been properly performed. For himself he concludes that (1) the reaction is of great diagnostic value in typhoid fever; (2) if the case shows a slight or no reaction between the fifth and eighth days of illness, other appearances pointing to typhoid fever, it can be looked upon at once as an exceedingly light form and the prognosis accordingly made; (3) gastro-intestinal catarrhs accompanied by fever never show the reaction; (4) very marked and persistent reactions may occur in mild cases of typhoid and do not justify a bad prognosis; (5) reactions appearing continuously (for several months) in cases of phthisis pulmonalis always indicates a grave prognosis.

The reagent is made up as follows: (*a*) Two grammes of sulphanilic acid; 50 c. c. of hydrochloric acid; 1000 e. c. of distilled water; (*b*) A one-half of one per cent solution of sodium nitrite ($NaNO_2$). Fifty parts of *a* and one part of *b* are mixed, and equal parts of this reagent and of urine are placed in a test-tube and saturated with ammonia. Where the reaction is positive a carmine-red color is assured, which must also be visible in the foam. The reaction is based on the formation of diazo-sulphobenzol by the action of the nitrous acid on sulphanilic acid. This acting on certain aromatic bodies in the urine produces the aniline colors on which the recognition of the reaction depends. A paper on the same subject also strongly supporting the claim for the value of this test in the diagnosis of typhoid fever is published by Prof. C. L. Greene, of the University of Minnesota (*Northwestern Lancet*, Jan. 1, 1894).

---

NEUROSIS FOLLOWING ENTERIC FEVER.--Dr. William Osler, of Baltimore (*Amer. Jour. of Med. and Science*, January, 1894), publishes under this caption the records of several cases of what has been previously described under the name of "typhoid spine," a painful condition of the back following attacks of typhoid fever, and regarded by many authorities as evidence of a spondylitis or perispondylitis of the vertebræ. In all the cases mentioned by Osler there appeared a more or less pronounced condition of neurasthenia, and the writer is disposed to regard the painful condition as a neurotic manifestation analogous to the well-known "hysterical spine." He admits the possibility, however, of existence in some cases of actual structural foundation, which might be of the nature of a periostitis, such as occurs not very rarely about the sternum and ribs; or it might possibly be the outcome of actual disease of the cord.

---

THE TREATMENT OF PHTHISIS.—(*N. Y. Med. Record*, Dec. 23, 1803.) There has recently appeared from the pen of Dr. C. E. Hershey, C. E., of Denver, Col., an excellent article upon the treatment of consumption. The tone of the paper is iconoclastic, but nevertheless the substance is highly credible and most applicable at the present time of therapeutic vagrancy. The author names some of the most lauded measures suggested for the cure of pulmonary phthisis within the past decade,—inhalation of compressed air, inhalation of hot air, Bergeon's rectal injections of sulphuretted hydrogen gas, Koch's tuberculin, the much advertised chemical cure from Ohio,—and holds forth as the results of their employment—little or nothing! He insists on the futility and fallacy of the polypharmacy practiced so often and so persistently in these cases; the destruction of the integrity of the gastric function by pushing this, that and the other drug to its tolerance; the irritation of the lungs by various inhalations hoped to be curative; now a dose of quinine, now of acetanilide; phenacetine or antipyrine; presently a night-cap of opium or belladonna, or both; the almost unbroken exclusion of the patient from good, pure, fresh air—and after all this what may be expected but consumption? He insists on the only reasonable treatment—exercise, diet, fresh air, attendance upon the integrity of the functions of the body. Patients coming to such a climate as Colorado do well in proportion as they have not been subjects of the hap-hazzard present-day dosage by their physicians. Dr. Mershey indicates the meaning of this statement in a tabular way from his own experience: Of 92 cases coming under his notice after two to five years active treatment by the ordinary methods of the day, over 66 per cent have proved fatal; of 90 treated from one to two years, more than 35 per cent have been fatal; of 41 treated from three months to a year, nearly 20 per cent have proved fatal; while of 97 having had no previous treatment, a little more than 5 per cent only have proved fatal. "No cure has as yet been accomplished by attempts to destroy tubercle bacilli, and there is every reason to believe that much harm may be done by taking away the excellent advantages afforded by nature to eradicate the disease." He quotes Alonzo Clark's treatment as surpassing in excellence: "Consumption may be cured in its early stages. Horse-back exercise, twenty-five miles daily; one to one and a half quarts of milk, with half a pint of cream daily; rare beef; friction to surface of body night and morning; peaches; in case of poor appetite, a teaspoonful of tr. gentian co. in a good sherry wine three times daily: and in case of severe cough, spray the throat with glycerine and water, a grain of opium to the ounce." The entire article is timely and sensible, and loses much by being abstracted.

---

HOT WATER IN ACUTE ALCOHOLISM.—Burson (*North American Practitioner*, April, 1893) presents a series of eight cases of acute alcoholism treated by the administration of hot water in cupful doses, every hour, or more or less frequently. They were all males, and all gave a history of habitual alcoholism. One case was complicated with contusions; one with a fracture of the tibia; two with pneumonia; one with la grippe, and one with a traumatic hæmothorax. One had been given bromide of potass. (30 gr.) and choral (15 gr.) every hour for twenty-four hours, and one bromide (30 gr.) alone for five hours, with little sedative effect; and another had been given small amounts of whisky for three days without appreciable influence. All were confined to bed; some were restrained forcibly, and all received a cupful of hot milk every two hours as nourishment. The delirium averaged from two to three days after the treatment had been started, improvement beginning shortly after the beginning of the treatment. The author holds hot water to be decidedly indicated to relieve the inflammation of the gastric mucus membrane; to wash away the mucus covering it; to increase the inter-arterial blood pressure, and diminish the cardiac rate which have both been modified by the alcohol, as well as to quiet the nervous system and soothe the kidneys. It is also of great aid in removing the alcohol present, mixture being readily formed between warm water and alcohol.

---

DISINFECTION IN AN EPIDEMIC OF PNEUMONIA.—Dr. Jerome Cochran, health officer of the State of Alabama (*Alabama Medical and Surgical Age*, November, 1893), states that at the prison at Pratt's Mines, Alabama, known as prison No. 2, there recently occurred an epidemic of well-marked pneumonia, strictly confined to this prison, no cases appearing in another prison building (Prison No. 1), about one mile distant, or in Pratt City, two miles away. In prison No. 2 there was a population of about six hundred persons. The epidemic began in February last with three cases; in April there were 22 cases; in May 65 cases, distributed as follows: First week, 14 cases; second week, 31 cases; third week, 13 cases; the remainder of the month, 7 cases, ending the epidemic. In all there were 93 cases; the total number of deaths were 30. From its marked prevalence in this detached community it was readily recognized as a distinct infectious disease, and efforts toward disinfection were begun on the last day of the second week in May (the height of the epidemic). The prison consisted of three sections. While one section was being disinfected, the convicts were crowded into the other two sections. Then the mattresses were removed from the bunks, as well as the blankets and mattress cases, and thrown about the floor. After this, by a force pump and long hose, the ceiling, walls and floors, with their contents, were flooded with a 1-1000 solution of bichloride of mercury, until it stood in puddles and ran in rivulets over the floors. The bedding was wetted as thoroughly as possible, then, for fear of non-penetration by the mercurial solution, it was placed in steam chambers and steamed for six hours, then dried. The wards had, by this time, been thoroughly scrubbed, white-washed and dried; and the convicts, after having taken a bath, and put on clean clothes, were returned. The same measures were then carried out in the other sections. As the cases arose, of course they were removed from the sections and isolated for treatment. In the same journal Dr. P. M. Cunningham refers to the same epidemic (*Report of Tri-State Medical Society Transactions*), in whose paper it is stated that the morbility of this epidemic was especially marked among negroes, perhaps twice the number of blacks becoming affected as of whites.

---

THE TREATMENT OF TETANUS.—Dr. George H. Lee, of Galveston (*New Orleans Medical and Surgical Journal*, July, 1893), has lately contributed an interesting article upon the influence of eserine in the treatment of tetanus, detailing five cases, in four of which recovery followed the employment of this agent. Dr. Lee indicates the following line of treatment: (1) The focus of bacterial growth, in which the active toxic agents are being developed, is to be removed or destroyed. Where amputation is conservatively possible, it should be performed to get rid of this focus; otherwise the thorough curetting of the sore, or the free incision into inflamed or suppurating foci, with application of germicides, is indicated. Of the antiseptics, bichloride of mercury, iodol, iodoform and aristol, as well as others, have been suggested. The writer suggests the possible value of peroxide of hydrogen, because of the well known anaerobic character of the tetanus micro-organisms. He has, however, used in all his cases, and with no bad effects, free applications of bichloride of mercury. (2) The effects of the toxines upon the general system must be met by antidotes whose physiological effects counteract the symptoms produced by the poisons, just as a case of strychnine or other poisoning would be treated. In the search for such an antidote Dr. Lee reasons that from the congestive and other changes found at autopsy, in the spinal cord and medulla, in cases of tetanus, it is to be supposed that the tonic convulsions of the disease are the result of rapidly-repeated nerve waves originating in an exalted irritability of the reflex centres of these parts. There is no evidence that direct action of the toxines upon the muscular system has any influence in inducing these contractions. The desired antidote is therefore to be sought among the spinal depressants; and chloral, the bromides and the opiates are well known to have acted favorably in many instances. Chloral and the bromides, however, lose some of their value from their cerebral influences, in that they may induce comatose conditions in which there is likelihood of interference with nutrition; and opium is objectionable in this or that case, if not in all, because of its interference with secretion and excretion. In Dr. Lee's opinion physostigma affords the most satifactory antidode to the poison of tetanus, particularly its alkaloid eserine. This alkaloid he would give hypodermically in doses of one-twentieth of a grain every four hours, gradually but quickly increasing to one-sixth of a grain, or until the jaws are relaxed and the patient is resting easily. The author records one case among those forming the basis of his paper, in which the drug was badly borne, convulsions invariably following its administration. This untoward result he would attribute either to a personal indiosyncrasy of his patient, to possible impurity of the drug, or to a possible paralysis of the inhibitory centres of the cord. As a rule, however, the effect of the drug was only a favorable one in the writer's experience, evident to the patient as well as to the attendants and the physician. (3) The removal of all possibility of external irritation from noise, light, draughts, etc. (4) The maintenance of nutrition by concentrated fluid food, peptonized milk, meat juice, beef and chicken broths, with whisky or brandy, as indicated. These should be given in full amounts, at regular intervals, by the mouth, or by enemata for short times, to bridge over crises.

